# Differential Influences of Endogenous and Exogenous Sensory Neuropeptides on the ATP Metabolism by Soluble Ectonucleotidases in the Murine Bladder Lamina Propria

**DOI:** 10.3390/ijms242115650

**Published:** 2023-10-27

**Authors:** Alejandro Gutierrez Cruz, Mafalda S. L. Aresta Branco, Mahsa Borhani Peikani, Violeta N. Mutafova-Yambolieva

**Affiliations:** Department of Physiology and Cell Biology, School of Medicine, University of Nevada Reno, Reno, NV 89557, USA; agutierrezcruz@unr.edu (A.G.C.); mborhanipeikani@unr.edu (M.B.P.)

**Keywords:** bladder, neuropeptides, ATP, ectonucleotidases, urothelium, CGRP, substance P, PACAP, neurokinin A, purinergic signaling

## Abstract

Bladder urothelium and suburothelium/lamina propria (LP) have prominent sensory and transducer functions with the active participation of afferent neurons and urothelium-derived purine mediators such as adenosine 5’-triphosphate (ATP), adenosine 5’-diphosphate (ADP), and adenosine (ADO). Effective concentrations of purines at receptor targets depend significantly on the extracellular degradation of ATP by ectonucleotidases (ENTDs). We recently reported the regulated release of soluble ENTDs (s-ENTDs) in the LP and the consequent degradation of ATP to ADP, AMP, and ADO. Afferent neurons in the LP can be activated by urothelial ATP and release peptides and other transmitters that can alter the activity of cells in their vicinity. Using a murine decentralized ex vivo detrusor-free bladder model, 1,N^6^-etheno-ATP (eATP) as substrate, and sensitive HPLC-FLD methodologies, we found that exogenous neuropeptides calcitonin gene-related peptide (CGRP), substance P (Sub P), neurokinin A (NKA), and pituitary adenylate cyclase-activating polypeptide [PACAP (1-38)] all increased the degradation of eATP by s-ENTDs that were released in the LP spontaneously and/or during bladder filling. Using antagonists of neuropeptide receptors, we observed that endogenous NKA did not modify the ATP hydrolysis by s-ENTDs, whereas endogenous Sub P increased both the constitutive and distention-induced release of s-ENTDs. In contrast, endogenous CGRP and PACAP (1-38) increased the distention-induced, but not the spontaneous, release of s-ENTDs. The present study puts forward the novel idea that interactions between peptidergic and purinergic signaling mechanisms in the LP have an impact on bladder excitability and functions by regulating the effective concentrations of adenine purines at effector cells in the LP.

## 1. Introduction

The bladder urothelium is a physical barrier that protects the deep layers of the bladder wall from direct contact with the content of the urine. The urothelium also senses distention of the bladder wall during bladder filling and releases biologically active mediators, such as adenosine 5′-triphosphate (ATP), in both the suburothelium/lamina propria (LP) and lumen [1]. In addition to mechanical forces [2,3,4], activation of membrane receptors and ion channels, cell swelling, and pH alterations cause the release of ATP at both the serosal/abluminal and mucosal/luminal sides of flat bladder mucosa sheets [5,6,7]. Released ATP activates multiple P2X and P2Y purinergic receptors in various cell types within the bladder wall, including urothelial cells, sensory neurons, interstitial cells, motor neurons, and smooth muscle cells [8]. One of the major activities of urothelial ATP is proposed to be the activation of P2X2/X3 receptors on sensory afferent termini at the LP-urothelium interface to initiate the voiding reflexes [8,9]. To achieve its biological activities, however, ATP must be in effective concentrations at its receptor sites. The extracellular levels of ATP depend on the amounts of released ATP as well as on its subsequent degradation to adenosine 5′-diphosphate (ADP), adenosine 5′-monophosphate (AMP), and adenosine (ADO) by a number of membrane-bound and soluble ectonucleotidases (ENTDs) [10,11].

In addition to urothelial cells, distention of the bladder wall during filling is also detected by afferent nerve fibers that are located at different layers of the bladder wall and are particularly dense at the LP-urothelium interface. Activation of mechanosensitive afferent fibers generates a sensation of bladder fullness and activates micturition [9,12]. These neurons exhibit immunoreactivity for various neuropeptides, including calcitonin gene-related peptide (CGRP), pituitary adenylate cyclase-activating polypeptide (PACAP), vasoactive intestinal peptide (VIP), substance P (Sub P), and neurokinin A (NKA), that can be released during bladder filling or in response to inflammation [13,14,15,16]. A single nerve fiber can express and release multiple neuropeptides in the interstitial space, and therefore cells in the urothelium and LP can be exposed to many neuropeptides simultaneously.

Neuropeptides exert their biological effects by binding to diverse G-protein-coupled receptors (GPCRs) and activating several signaling pathways. They are involved in muscle contraction, vasodilation, regulation of cell proliferation, nociception, immune and stress responses, and neurogenic inflammation. Neuropeptides that are released in the bladder wall appear to increase bladder afferent nerve activity and release ATP from urothelial cells [14,15]. Subsequently, fluctuations in extracellular levels of neuropeptides would alter the extracellular levels of ATP at its receptor sites. The levels, location, and receptor density of neuropeptides in the lower urinary tract (LUT) may be altered in LUT dysfunctions caused by bladder inflammation or spinal cord injuries, resulting in impaired bladder reflexes and dysfunctional voiding [15,17]. Conversely, neuropeptides that are released/secreted in excessive amounts during pathophysiological conditions are themselves regarded as potent initiators of inflammation due to their influences on vascular permeability and plasma protein extravasation [18]. Bladder inflammation and spinal cord injuries are also associated with increased levels of ATP in the bladder wall [19,20]. At least in part, increased extracellular ATP might be caused by increased secretion of neuropeptides.

Four families of ENTDs, including membrane-bound ectonucleoside triphosphate diphosphohydrolases (ENTPDs), ectonucleotide pyrophosphatase/phosphodiesterases (ENPPs), tissue-nonspecific alkaline phosphatase (TNAP), and 5′-nucleotidase (NT5E/CD73), are involved in the extracellular degradation of ATP to ADP, AMP, and ADO [10]. We have recently described a novel highly-regulated mechanosensitive mechanism of extracellular degradation of ATP in the bladder LP by soluble ENTDs (s-ENTDs) that are released in the LP spontaneously and in response to bladder wall distention during filling [11,21,22]. These specialized enzymes may be responsible for maintaining proper bladder excitability by ceasing the excitatory effects of ATP in the LP and accumulating ADO at cell surfaces. Distention-activated sensory afferents appear to regulate the degradation of ATP by s-ENTDs during bladder filling [21]. It is possible, therefore, that sensory neuropeptides regulate the effective concentrations of extracellular ATP at receptor sites not only by influencing the release of ATP as discussed above but also by affecting its degradation. For that reason, the present study was designed to investigate whether CGRP, Sub P, NKA, PACAP (1-38), PACAP (1-27), and VIP affect the local degradation of extracellular ATP in the bladder LP by s-ENTDs in nondistended or distended bladders. We also tested whether endogenous and exogenous neuropeptides influence the release of s-ENTDs (and hence ATP degradation) in similar or distinct manners.

## 2. Results

### 2.1. Role of CGRP in Spontaneous and Distention-Induced Release of s-ENTDs in the LP

#### 2.1.1. Exogenous CGRP Increases the Spontaneous but Not the Distention-Induced Hydrolysis of eATP by s-ENTDs

**Figure 1:**
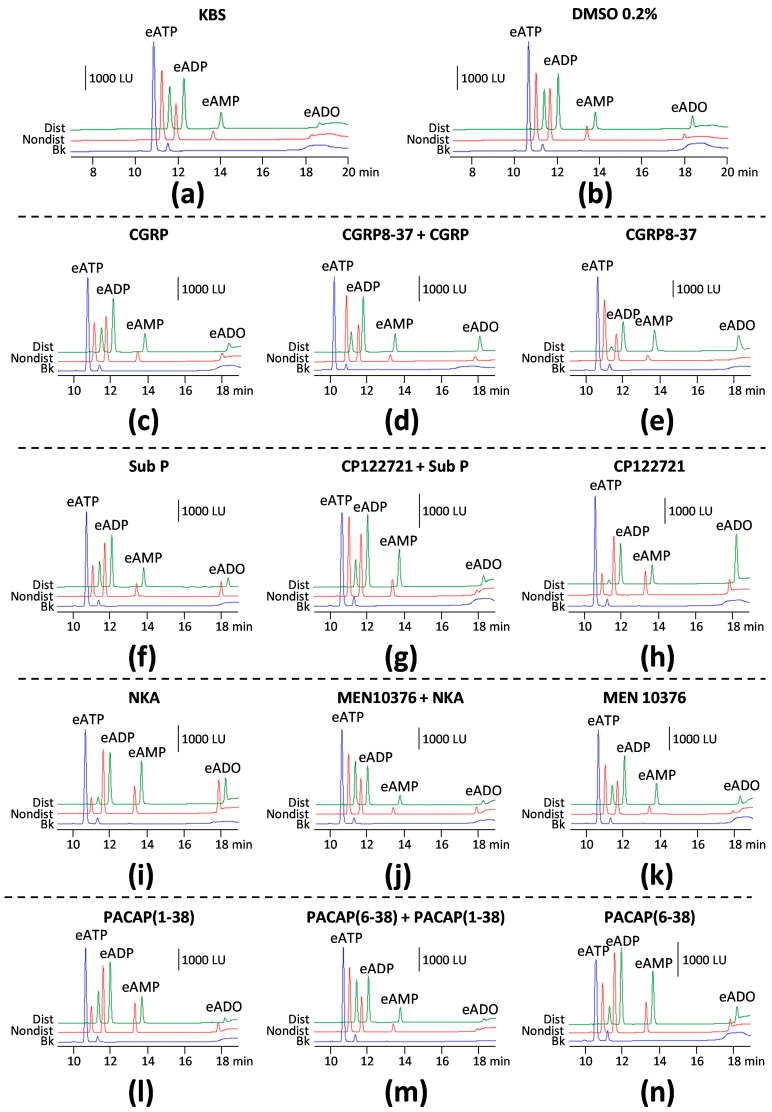
Representative original chromatograms demonstrating the degradation of eATP and formation of eADP, eAMP, and eADO by s-ENTDs released in cELS of nondistended (red) and distended (green) detrusor-free bladder preparations that were treated with vehicle [KBS, (**a**); DMSO 0.2%, (**b**)], exogenous neuropeptides [CGRP, 100 nM, (**c**); Sub P, 1 µM, (**f**); NKA, 100 nM, (**i**); and PACAP (1-38), 100 nM, (**l**)], exogenous neuropeptides in the presence of receptor antagonists [CGRP8-37, 1 µM + CGRP, 100 nM, (**d**); CP122721, 1 µM + Sub P, 1 µM, (**g**); MEN 10376, 10 µM + NKA 300 nM, (**j**); and PACAP (6-38), 300 nM + PACAP (1-38), 100 nM, (**m**)], and with antagonists of neuropeptide receptors [CGRP8-37, 1 µM, (**e**); CP122721, 1 µM, (**h**); MEN 10376, 10 µM, (**k**); and PACAP (6-38), 300 nM, (**n**)]. Blue, eATP substrate (no enzyme present). Note that in vehicle controls, the decrease in eATP and increase in e-products were greater in samples of distended preparations than in non-distended preparations. The decrease in eATP and increase in products vary in the cELS of bladder preparations treated with drugs. In cELS of bladder preparations treated with antagonist plus agonist, the degradation of eATP resembled the controls. The receptor antagonists CGRP8-37, CP122721, and PACAP (6-38) modified the degradation of eATP in cELS, suggesting that the endogenous agonists of these receptors (i.e., CGRP, Sub P, and PACAP (1-38) alter the release of s-ENTDs.

**Figure 2:**
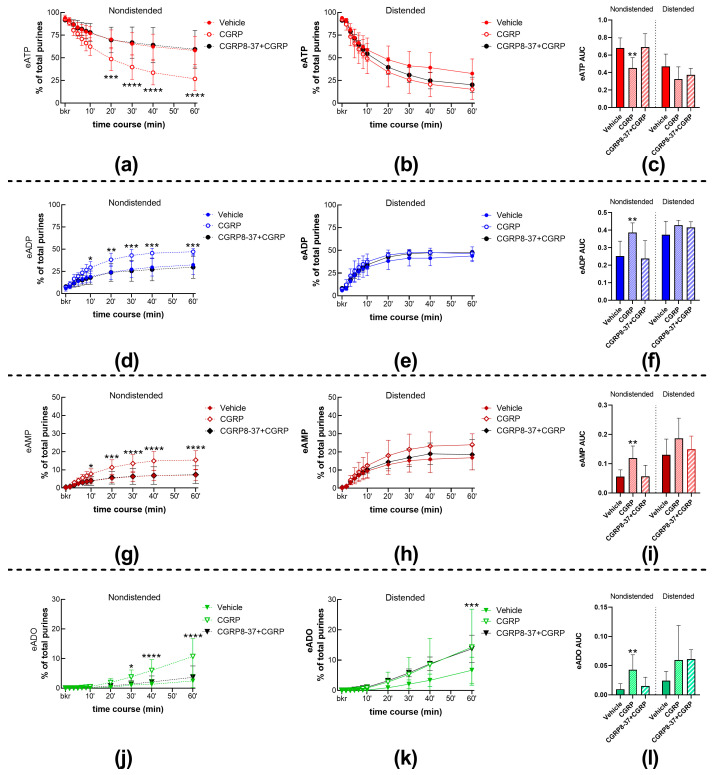
Summed data of time courses of eATP decrease (**a**,**b**) and increase in eADP (**d**,**e**), eAMP (**g**,**h**), and ADO (**j**,**k**) in cELS of nondistended (dashed lines) and distended (solid lines) preparations in the presence of vehicle (n = 9 nondistended, n = 8 distended), CGRP (n = 5), and CGRP8-37 plus CGRP (n = 4). n, number of observations. Each purine is represented as a percentage of total purines (eATP + eADP + eAMP + eADO) detected in reaction solutions at each time point. Asterisks denote significant differences from vehicle controls. * *p* < 0.05, ** *p* < 0.01, *** *p* < 0.001, **** *p* < 0.0001; two-way ANOVA with Sidak’s multiple comparisons test. Mean area under the curve (AUC) values for time-courses of eATP (**c**), eADP (**f**), eAMP (**i**), and eADO (**l**). Asterisks denote significant differences from vehicle controls. ** *p* < 0.01; ordinary one-way ANOVA with Dunnett’s multiple comparisons test within nondistended and distended bladder preparations.

To evaluate the effects of CGRP receptor activation on the degradation of eATP by s-ENTDs in the LP, we monitored the decrease in eATP and the increase/appearance of its products eADP, eAMP, and eADO in cELS of nondistended and distended denuded bladders treated with either vehicle (KBS, controls) or with 100 nM CGRP. Representative chromatograms of samples at 60 min of reaction between substrate eATP and released enzymes are shown in Figure 1. Thus, in both KBS (Figure 1a) and DMSO 0.2% (Figure 1b), there was a decrease in eATP and an increase or appearance of the eATP products eADP, eAMP, and eADO. The degradation of eATP was greater in cELS samples of nondistended bladders than distended bladders. Compared with KBS control, exogenous CGRP (Figure 1c) increased the degradation of eATP in cELS of nondistended bladder preparations, which resulted in eATP being similar in samples from nondistended and distended bladders. Summed data shown in Figure 2 demonstrate that CGRP significantly accelerated the decrease in eATP at 20–60 min of enzymatic reaction (Figure 2a) and the formation of eADP (Figure 2d, 10–60 min of reaction), eAMP (Figure 2g, 10–60 min of reaction), and eADO (Figure 2j, 30–60 min of reaction) when compared with the effects of vehicle (KBS) in cELS of nondistended bladder preparations. The degradation of eATP in cELS of distended preparations largely remained unchanged by CGRP (Figure 2b,e,h), with the exception of the eADO formation, which was significantly increased at 60 min of reaction only (Figure 2k). Similar results were obtained when the decrease in eATP and increase in e-products were analyzed by AUC (Figure 2c,f,i,l). Thus, in cELS of nondistended bladders, the AUC of eATP was significantly decreased, whereas the formation of eADP, eAMP, and eADO was significantly increased. In cELS of distended preparations, the AUC of eATP substrate and e-products was not significantly different from the AUC of each purine in cELS of preparations treated with vehicle. 

#### 2.1.2. The Effect of CGRP on Spontaneous s-ENTD Release Is Mediated by Receptors Sensitive to CGRP8-37 

As shown in Figure 2a,d,g,j, the CGRP receptor antagonist CGRP8-37 (1 µM) [23] abolished the increasing effect of CGRP (100 nM) on eATP degradation in cELS of nondistended bladder preparations. Pretreatment with CGRP8-37 did not change the lack of effect of CGRP on eATP metabolism in cELS from distended preparations (Figure 2b,e,h, k). CGRP in the presence of CGRP8-37 showed no statistically significant effects on s-ENTD release during a 1-h time course of enzymatic reactions (Figure 2a,b,d,e,g,h,j,k), which was also seen in the AUC (Figure 2c,f,i,l) of each purine. The representative chromatograms in Figure 1d show a lack of increased degradation of eATP by CGRP when the preparation was pretreated with CGRP8-37 when compared with the KBS control in Figure 1a. These results suggest that the increasing effect of CGRP on spontaneous s-ENTD release is mediated by a CGRP8-37-sensitive CGRP receptor.

#### 2.1.3. Inhibition of CGRP Receptors with CGRP8-37 Increased the Distention-Induced Release of s-ENTDs but Not the Spontaneous Release of Enzymes

**Figure 3:**
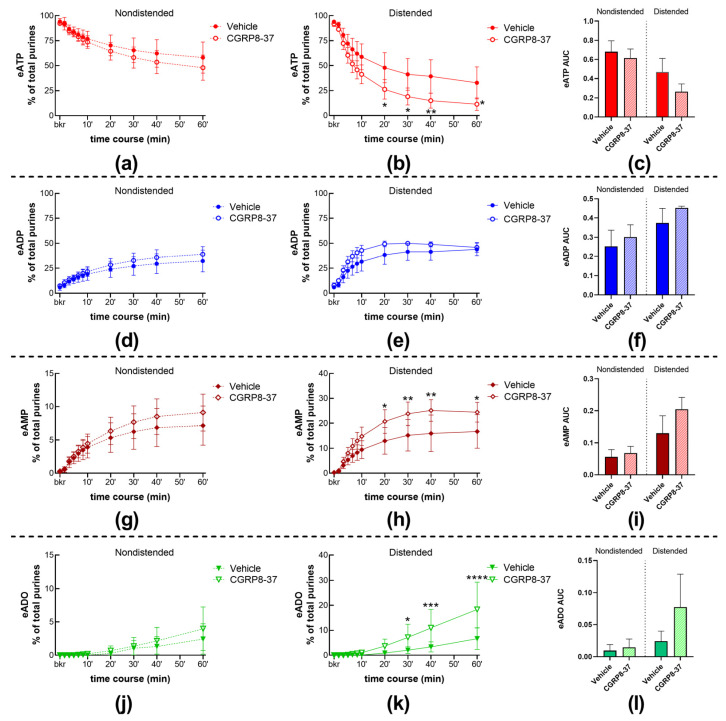
Summed data of time courses of eATP decrease (**a**,**b**) and increase in eADP (**d**,**e**), eAMP (**g**,**h**), and ADO (**j**,**k**) in cELS of nondistended (dashed lines) and distended (solid lines) preparations in the presence of vehicle (n = 9 nondistended, n = 8 distended) and CGRP8-37 (n = 4). n, number of observations. Each purine is represented as a percentage of total purines (eATP + eADP + eAMP + eADO) detected in reaction solutions at each time point. Asterisks denote significant differences from vehicle controls. * *p* < 0.05, ** *p* < 0.01, *** *p* < 0.001, **** *p* < 0.0001; two-way ANOVA with Sidak’s multiple comparisons test. Mean area under the curve (AUC) values for time-courses of eATP (**c**), eADP (**f**), eAMP (**i**), and eADO (**l**). *p* > 0.05 from vehicle controls, unpaired t-test.

CGRP8-37 alone had no significant effect on the spontaneous release of s-ENTDs, as demonstrated by the lack of significant alterations in eATP decrease or eADP, eAMP, and eADO increase (Figure 1a,e; Figure 3a,d,g,j). However, the degradation of eATP and the formation of its e-products eADP, eAMP, and eADO were increased in cELS of distended preparations treated with CGRP8-37 (Figure 1a,e; Figure 3b,e,h,k). The effects of CGRP8-37 reached statistical significance at 20–60 min of reaction for eATP and eAMP, whereas the increase in eADO was significantly higher at 30–60 min of reaction. The AUC of eATP decrease and e-product increase suggested increased degradation of eATP; however, statistical significance was not reached (Figure 3c,f,i,l).

**Figure 4:**
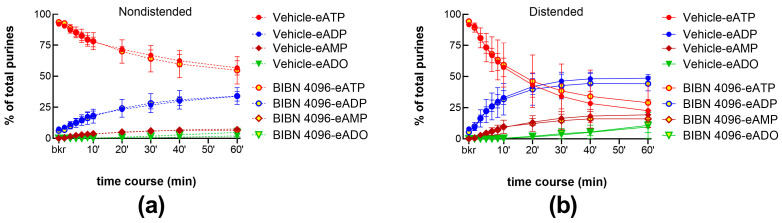
eATP degradation by s-ENTDs released in cELS of nondistended (**a**) and distended (**b**) preparations in the presence of vehicle controls (DMSO 0.2%, n = 6) or BIBN 4096 (1 µM, n = 3). *p* > 0.05 from vehicle control; two-way ANOVA with Sidak’s multiple comparisons test.

In a separate set of experiments, we examined the degradation of eATP in cELS of bladder preparations treated with another CGRP receptor antagonist, BIBN 4096 (1 µM). The decrease in eATP and formation of e-products were not altered by this antagonist, neither in cELS of nondistended bladder preparations (Figure 4a) nor in cELS of distended bladder preparations (Figure 4b).

### 2.2. Role of the NK1 Receptor in Spontaneous and Distention-Induced Release of s-ENTDs in the LP

#### 2.2.1. Exogenous Substance P Increases the Spontaneous but Not the Distention-Induced Hydrolysis of eATP by s-ENTDs

To determine whether activation of NK1 receptors in the LP regulates the release of s-ENTDs, we examined the degradation of eATP in cELS of nondistended and distended detrusor-free bladder preparations treated with Sub P (1 µM), an agonist of NK1 receptors.

**Figure 5:**
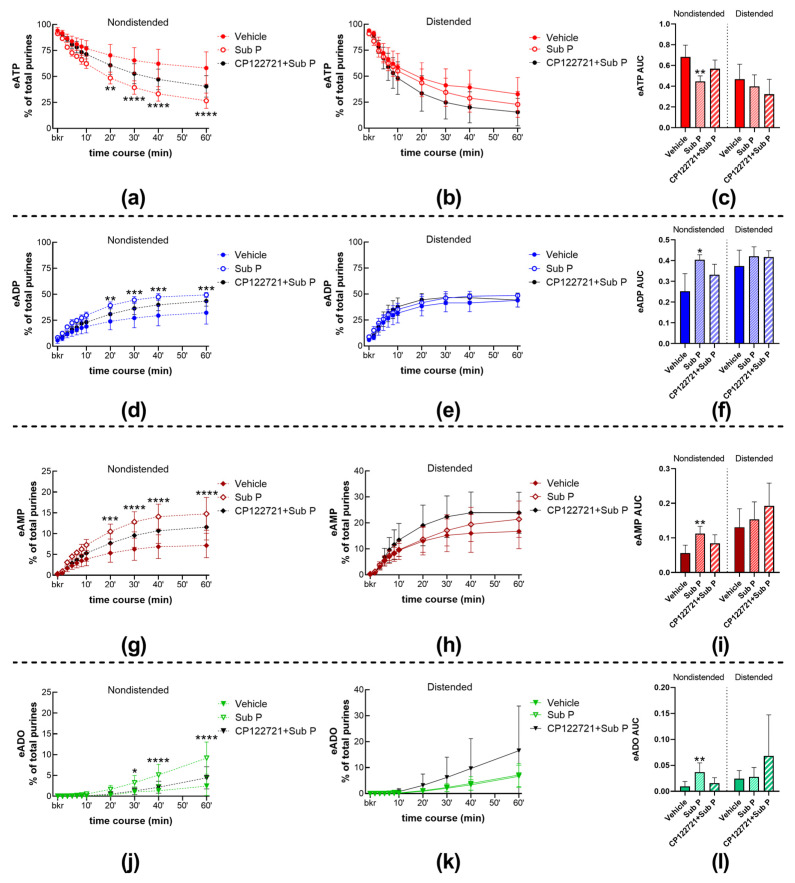
Summed data of time courses of eATP decrease (**a**,**b**) and increase in eADP (**d**,**e**), eAMP (**g**,**h**), and ADO (**j**,**k**) in cELS of nondistended (dashed lines) and distended (solid lines) preparations in the presence of vehicle (n = 9 nondistended, n = 8 distended), Sub P (n = 4), and CP122721 plus Sub P (n = 4). n, number of observations. Each purine is represented as a percentage of total purines (eATP + eADP + eAMP + eADO) detected in reaction solutions at each time point. Asterisks denote significant differences from vehicle controls. * *p* < 0.05, ** *p* < 0.01, *** *p* < 0.001, **** *p* < 0.0001; two-way ANOVA with Sidak’s multiple comparisons test. Mean area under the curve (AUC) values for time-courses of eATP (**c**), eADP (**f**), eAMP (**i**), and eADO (**l**). Asterisks denote significant differences from vehicle controls. * *p* < 0.05, ** *p* < 0.01; ordinary one-way ANOVA with Dunnett’s multiple comparisons test within nondistended and distended bladder preparations.

The decrease in eATP and increase in eADP, eAMP, and eADO (Figure 1a,f; Figure 5a,d,g,j) were significantly increased in samples of nondistended bladders treated with Sub P. Statistical significance was reached in eATP, eADP, and eAMP changes at 20–60 min of reaction and at 30–60 min of reaction for eADO. However, the decrease in eATP and increase in e-products were not modified in the cELS of distended preparations treated with Sub P (Figure 1a,f; Figure 5b,e,h,k). These observations were made in both the time-course of the enzymatic reaction and the AUC (Figure 5c,f,i,l) of substrate decrease and product increase during one hour of the enzymatic reaction.

The increasing effect of Sub P on the degradation of eATP in cELS of nondistended preparations was diminished in the presence of the selective antagonist of NK1 receptors, CP122721 (1 µM) [24] (Figure 1a,g; Figure 5a,d,g,j), whereas the degradation of eATP in cELS of distended preparations treated with CP122721 was not significantly different from controls (Figure 1a,g; Figure 5b,e,h,k). Similar effects were observed when the AUC was plotted (Figure 5c,f,i,l).

#### 2.2.2. The NK1 Receptor Inhibitor CP122721 Increases eATP Hydrolysis in the LP of Nondistended and Distended Bladders 

**Figure 6:**
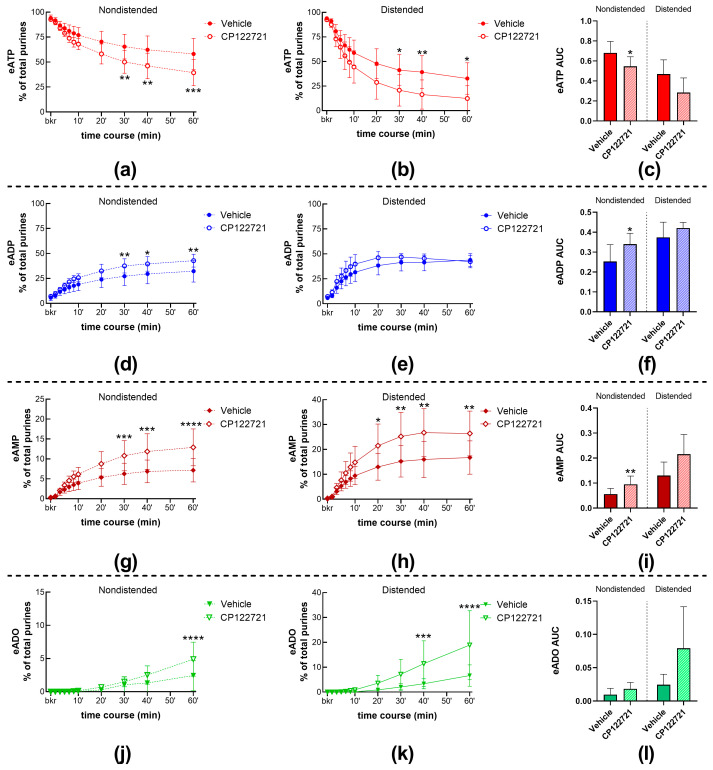
eATP degradation by s-ENTDs released in cELS of murine ex vivo detrusor-free bladder preparations in the presence of vehicle (KBS) and CP122721 (1 µM). Summed data of time courses of eATP decrease (**a**,**b**) and increase in eADP (**d**,**e**), eAMP (**g**,**h**), and ADO (**j**,**k**) in cELS of nondistended (dashed lines) and distended (solid lines) preparations in the presence of vehicle (KBS, n = 9 nondistended, n = 8 distended) and CP122721 (n = 7). n, number of observations. Each purine is represented as a percentage of total purines (eATP + eADP + eAMP + eADO) detected in reaction solutions at each time point. Asterisks denote significant differences from vehicle controls. * *p* < 0.05, ** *p* < 0.01, *** *p* < 0.001, **** *p* < 0.0001; two-way ANOVA with Sidak’s multiple comparisons test. Mean area under the curve (AUC) values for time-courses of eATP (**c**), eADP (**f**), eAMP (**i**), and eADO (**l**). * *p* < 0.05, ** *p* < 0.01; ordinary one-way ANOVA with Dunnett’s multiple comparisons test within nondistended and distended bladder preparations.

The decrease in eATP and increase in eADP, eAMP, and eADO were significantly potentiated in cELS of nondistended (Figure 1a,h; Figure 6a,d,g,j) bladders treated with CP122721 (1 µM), a non-peptide antagonist of the NK1 receptor. This was observed in both the time course of reactions and the AUC (Figure 6c,f,i,l-left panels) of eATP, eADP, and eAMP. The degradation of eATP and formation of eADP, eAMP, and eADO were also increased in cELS of distended preparations treated with CP122721 (Figure 1h); however, statistically significant effects were observed in the time course of enzymatic reactions (Figure 6b,h,k) but not in the AUC (Figure 6c,f,i,l-right panels). 

### 2.3. Role of the NK2 Receptor on Spontaneous and Distention-Induced Release of s-ENTDs in the LP

#### 2.3.1. The NK2 Receptor Agonist NKA Increases eATP Hydrolysis in the LP of Nondistended and Distended Bladders 

**Figure 7:**
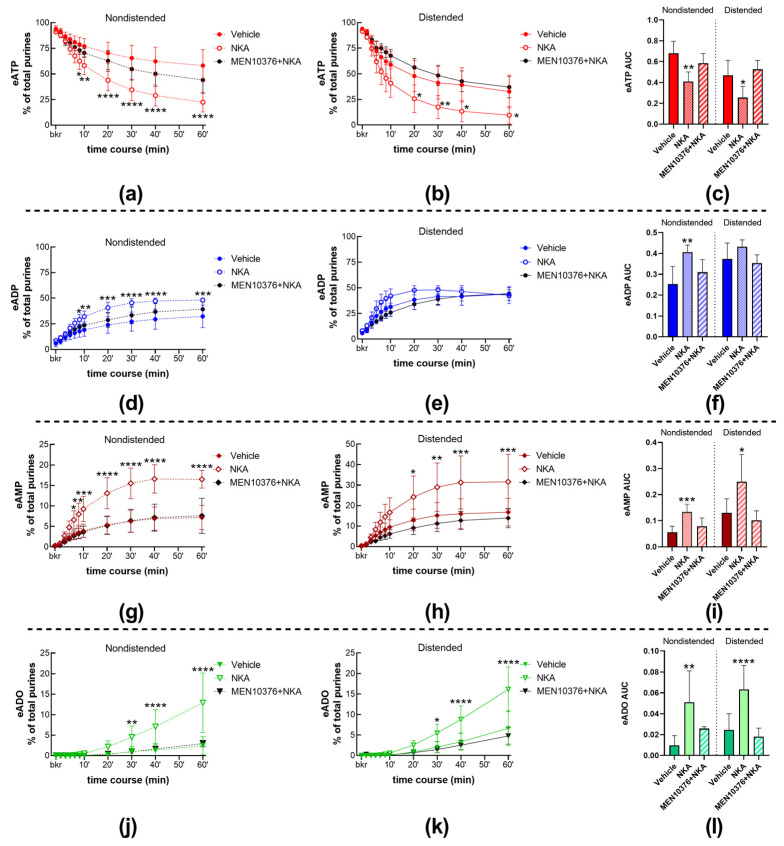
Summed data of time courses of eATP decrease (**a**,**b**) and increase in eADP (**d**,**e**), eAMP (**g**,**h**), and ADO (**j**,**k**) in cELS of nondistended (dashed lines) and distended (solid lines) preparations in the presence of vehicle (n = 9 nondistended, n = 8 distended), NKA (n = 4), and MEN 10376 plus NKA (n = 3). n, number of observations. Each purine is represented as a percentage of total purines (eATP + eADP + eAMP + eADO) detected in reaction solutions at each time point. Asterisks denote significant differences from vehicle controls. * *p* < 0.05, ** *p* < 0.01, *** *p* < 0.001, **** *p* < 0.0001; two-way ANOVA with Sidak’s multiple comparisons test. Mean area under the curve (AUC) values for time-courses of eATP (**c**), eADP (**f**), eAMP (**i**), and eADO (**l**). Asterisks denote significant differences from vehicle controls. * *p* < 0.05, ** *p* < 0.01, *** *p* < 0.001, **** *p* < 0.0001; ordinary one-way ANOVA with Dunnett’s multiple comparisons test within nondistended and distended bladder preparations.

To determine whether the NK2 receptors are involved in the regulation of eATP degradation by s-ENTDs in the LP, we next measured the decrease in eATP and increase in eADP, eAMP, and ADO in cELS of nondistended and distended preparations treated with either vehicle (KBS) or 100 nM NKA. NKA significantly increased the degradation of eATP in both cELS of nondistended and distended bladders. Thus, the decrease in eATP was accelerated at 8–60 min of reaction in cELS of nondistended preparations (Figure 7a) and at 20–60 min of reaction in cELS of distended preparations (Figure 7b). NKA treatment also resulted in increased formation of eADP, eAMP, and eADO in cELS of nondistended (Figure 7d,g,j) and distended (Figure 6e,h,k) bladders. The enhancing effect of NKA on eATP degradation was seen in both the time course of reactions as well as in the AUC of individual purines. The increasing effect of exogenous NKA on eATP degradation in cELS of nondistended and distended preparations is also illustrated in representative chromatograms in Figure 1i when compared with KBS control (Figure 1a).

The enhancing effect of NKA on eATP degradation in cELS of both nondistended (Figure 1a,j; Figure 7a,d,g,j) and distended (Figure 1a,j; Figure 7b,e,h,k) preparations was inhibited by the specific NK2 receptor antagonist MEN 10376 [25] at a concentration of 10 µM, suggesting that the observed effects of NKA were mediated by NK2 receptors that were sensitive to MEN 10376. 

#### 2.3.2. The NK2 Receptor Antagonist MEN 10376 Does Not Affect the Spontaneous or Distention-Induced Release of s-ENTDs in the LP

**Figure 8:**
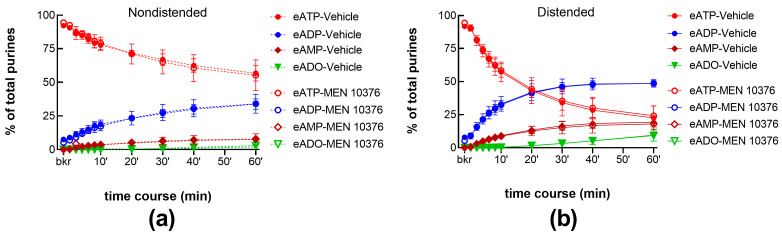
eATP degradation by s-ENTDs released in cELS of nondistended (**a**) and distended (**b**) preparations in the presence of vehicle (DMSO 0.2%, n = 6) or MEN 10376 (10 µM, n = 3). *p* > 0.05 from vehicle control; two-way ANOVA with Sidak’s multiple comparisons test.

To determine whether endogenously released NKA can affect the release of s-ENTDs in the LP, we evaluated the degradation of eATP and formation of eADP, eAMP, and eADO in cELS of bladders treated with either MEN 10376 (10 µM) or its vehicle (DMSO 0.2%). MEN 10376 failed to alter the degradation of eATP in cELS of nondistended (Figure 8a) or distended (Figure 8b) bladders. This is also illustrated with representative chromatograms in Figure 1k when compared with a vehicle (DMSO 0.2%) control (Figure 1b). These results suggest that endogenous NKA may not be involved in the regulation of s-ENTD release in the LP.

### 2.4. Role of the PAC1 Receptor in s-ENTD Release in the LP

#### 2.4.1. Exogenous PACAP (1-38) Increases the Distention-Induced Release of s-ENTDs but Not the Spontaneous Release of Enzymes in the LP

**Figure 9:**
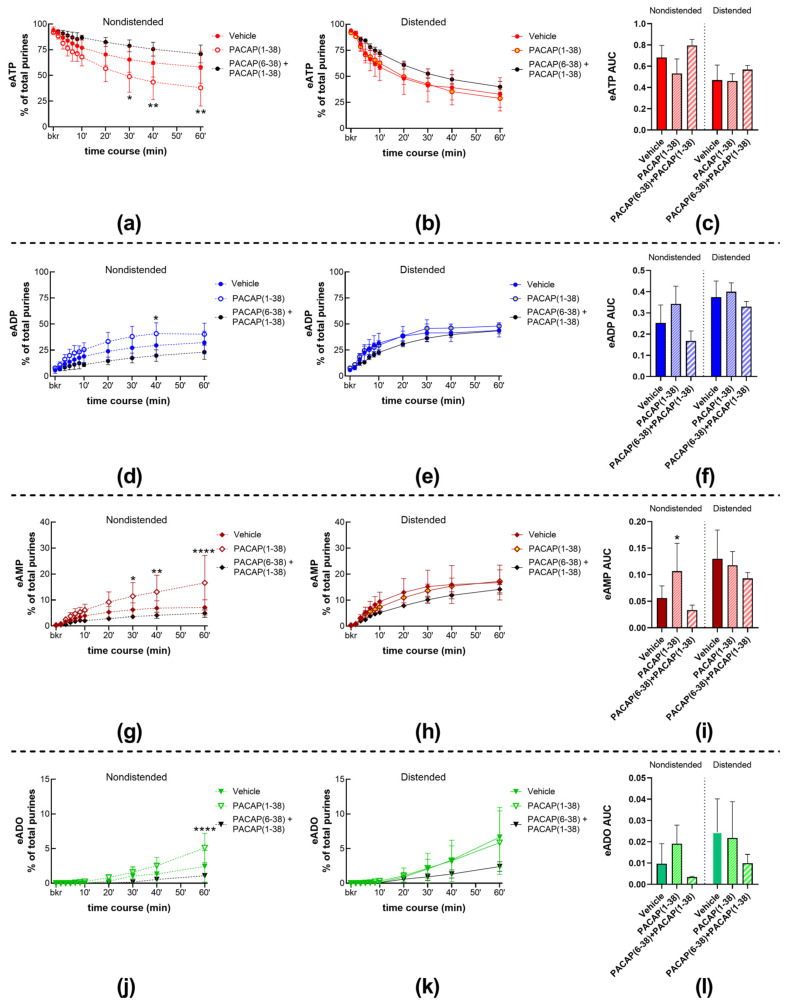
eATP degradation by s-ENTDs released in cELS of murine ex vivo detrusor-free bladder preparations in the presence of vehicle (KBS), PACAP (1-38) (100 nM), and PACAP (6-38) (300 nM) plus PACAP (1-38) (100 nM). Summed data of time courses of eATP decrease (**a**,**b**) and increase in eADP (**d**,**e**), eAMP (**g**,**h**), and ADO (**j**,**k**) in cELS of nondistended (dashed lines) and distended (solid lines) preparations in the presence of vehicle (KBS, n = 9 nondistended, n = 8 distended), PACAP (1-38) (n = 5), and PACAP (6-38) plus PACAP (1-38) (n = 3). n, number of observations. Each purine is represented as a percentage of total purines (eATP + eADP + eAMP + eADO) detected in reaction solutions at each time point. Asterisks denote significant differences from vehicle controls. * *p* < 0.05, ** *p* < 0.01, **** *p* < 0.0001; two-way ANOVA with Sidak’s multiple comparisons test. Mean area under the curve (AUC) values for time-courses of eATP (**c**), eADP (**f**), eAMP (**i**), and eADO (**l**). * *p* < 0.05; ordinary one-way ANOVA with Dunnett’s multiple comparisons test within nondistended and distended bladder preparations.

To determine whether activation of PAC1 receptors with PACAP (1-38) affects the release of s-ENTDs, we evaluated the degradation of eATP in cELS of nondistended and distended bladders treated with 100 nM PACAP (1-38). The decrease in eATP and the increase in eAMP and eADO were greater in cELS of nondistended preparations treated with PACAP (1-38) than with vehicle (Figure 1a,l; Figure 9a,g,j). The decrease in eATP and increase in eAMP were significantly augmented at 30–60 min of reaction. eADP levels were significantly increased only at 40 min of reaction (Figure 9d), whereas eADO formation in cELS of PACAP (1-38)-treated bladders exceeded the eADO formation in cELS of vehicle (KBS)-treated bladders only at 60 min of reaction (Figure 9j). Changes in the AUC of each purine followed the same trend as the one observed in the time-course of enzymatic reactions and reached statistical significance for eAMP (Figure 9i). The degradation of eATP and formation of e-products in cELS of distended bladders were not significantly different between vehicle controls and PACAP (1-38) as demonstrated in the time courses of enzymatic reactions (Figure 9b,e,h,k), the AUC data (Figure 9c,f,i,l), and the representative chromatograms (Figure 1a,l).

Pretreatment of bladder preparations with 300 nM of the PAC1 inhibitor, PACAP (6-38) [26], eliminated the accelerating effect of PACAP (1-38) on eATP degradation in cELS of nondistended preparations (Figure 1a,m; Figure 9a,d,g,j). The degradation of eATP in cELS of distended preparations tended to be decreased when bladders were treated with PACAP (1-38) in the presence of PACAP (6-38), but statistical significance was not reached.

#### 2.4.2. Inhibition of the PAC1 Receptor with PACAP (6-38) Increased the Distention-Induced Release of s-ENTDs but Not the Spontaneous Release of s-ENTDs in the LP

**Figure 10:**
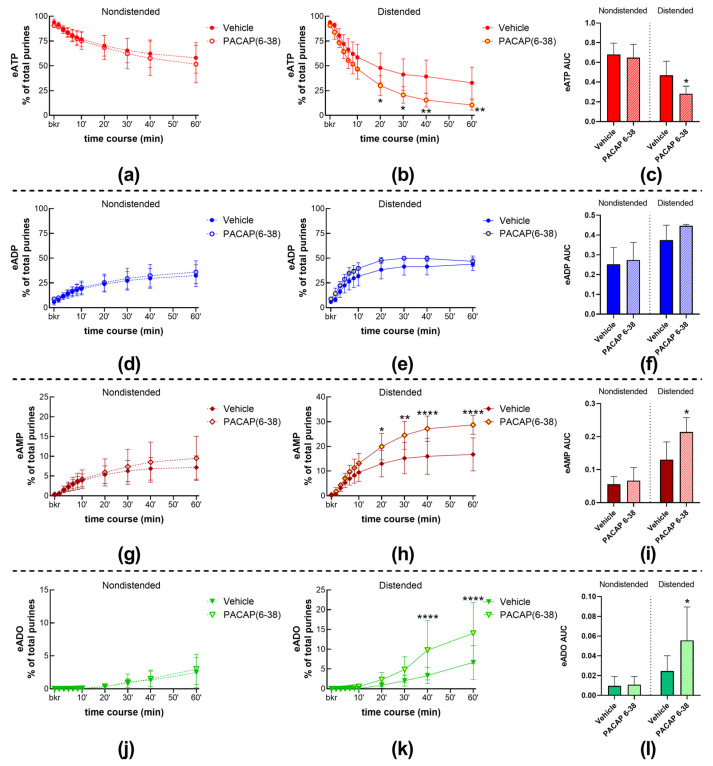
Summed data of time courses of eATP decrease (**a**,**b**) and increase in eADP (**d**,**e**), eAMP (**g**,**h**), and ADO (**j**,**k**) in cELS of nondistended (dashed lines) and distended (solid lines) preparations in the presence of vehicle (KBS, n = 9 nondistended, n = 8 distended) and PACAP (6-38) (n = 5). n, number of observations. Each purine is represented as a percentage of total purines (eATP + eADP + eAMP + eADO) detected in reaction solutions at each time point. Asterisks denote significant differences from vehicle controls. * *p* < 0.05, ** *p* < 0.01, **** *p* < 0.0001; two-way ANOVA with Sidak’s multiple comparisons test. Mean area under the curve (AUC) values for time-courses of eATP (**c**), eADP (**f**), eAMP (**i**), and eADO (**l**). Asterisks denote significant differences from vehicle controls. * *p* < 0.05; ordinary one-way ANOVA with Dunnett’s multiple comparisons test within nondistended and distended bladder preparations.

To assess whether activation of the PAC1 receptor with endogenous neuropeptides regulates the release of s-ENTDs in the LP, we determined the decrease in eATP and increase in e-products in cELS of nondistended and distended preparations treated with the PAC1 inhibitor PACAP (6-38). The degradation of eATP in cELS of nondistended preparations remained unchecked in the presence of PACAP (6-38) (time course Figure 10a,d,g,j; AUC Figure 10c,f,i,l-left panels). However, the degradation of eATP in cELS of distended bladders treated with PACAP (6-38) was augmented at 20–60 min of reaction for eATP and eAMP and at 40–60 min of reaction for eADO (Figure 10b,c,h,i). Representative chromatograms demonstrate that PACAP (6-38) increased the degradation of eATP in cELS of distended but not of nondistended preparations (Figure 1a,n).

**Figure 11:**
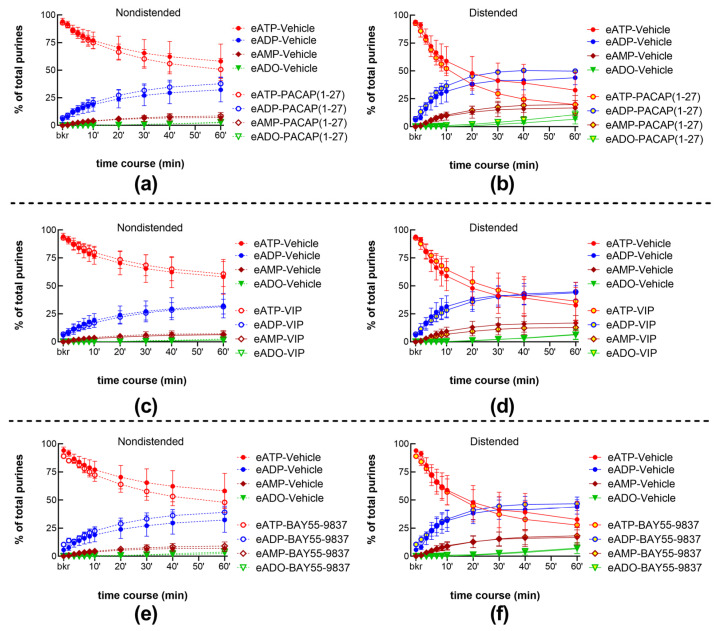
eATP degradation by s-ENTDs released in cELS of nondistended (dashed lines) and distended (solid lines) preparations in the presence of vehicle (KBS, n = 9 nondistended, n = 8 distended bladders) or PACAP (1-27) (100 nM, n = 3) (**a**,**b**), VIP (100 nM, n = 3) (**c**,**d**), and BAY 55-9837 (200 nM, n = 4) (**e**,**f**). *p* > 0.05 from vehicle control; two-way ANOVA with Sidak’s multiple comparisons test.

The peptides of the PACAP family, PACAP (1-27) 100 nM (Figure 11a,b) and VIP 100 nM (Figure 11c,d), and the VPAC2 receptor agonist BAY55-9837 100 nM (Figure 11e,f), had no effect on the spontaneous or distention-induced release of s-ENTDs.

## 3. Discussion

ATP is a primary regulator of bladder excitability due to its role in cell-to-cell communication and intracellular energy transfer. Once released from cells in the urothelium into the LP, ATP activates receptors on cell surfaces and stimulates afferent neurons and other cell types deep in the bladder wall. Subsequent ATP hydrolysis by ENTDs either terminates the ATP action or reassigns its biological activity to metabolites (e.g., ADP and ADO) that activate their own receptors on cell membranes. We have previously published that a part of extracellular ATP hydrolysis is performed by s-ENTDs released in the LP at rest and during bladder filling [11]. Inhibition of neural activity with tetrodotoxin or ɷ-conotoxin GVIA increased the distention-induced release but not the spontaneous release of s-ENTDs in the mouse bladder LP [21], suggesting possible regulation of s-ENTD release by sensory neuropeptides that might be secreted in the LP at rest or during bladder distention. Therefore, in the present study, we investigated the influences of a number of neuropeptides that are known to be present in the bladder wall as well as neuropeptide receptor antagonists on the hydrolysis of ATP by released s-ENTDs in the LP. We found that (1) multiple neuropeptides regulate the release of s-ENTDs, hence the extracellular ATP degradation in the LP, (2) the spontaneous and distention-induced release of s-ENTDs are not uniformly regulated by neuropeptides, (3) the release of s-ENTDs is affected differentially by endogenous and exogenous neuropeptides, (4) CGRP and PACAP (1-38) are putative endogenous mediators that restrain s-ENTD release, preventing potential excessive degradation of ATP at the end of bladder filling, and (5) endogenous Sub P likely participates in the fine regulation of bladder excitability by limiting the extracellular degradation of ATP to preserve proper purine concentrations at effector sites in both nondistended and distended bladders.

The general distribution of afferent fibers in rodent bladders has been commonly studied using CGRP-immunohistochemical staining along with classic fluorescence microscopy [12,27]. Afferent nerve fibers are distributed in both the LP and the detrusor muscle of the bladder wall. In the murine bladder LP, in particular, afferent nerve fibers are distributed throughout the entire LP, with the highest concentration of nerves, nerve branching, and end points at the interface between the LP and the urothelium [28]. Afferent nerves located in the LP detect information about the bladder filling status as well as pain signals and convey this information through the spinal cord to the responsible areas in the brain, affirming the role of the LP as a signal transducer and communication center [29]. Bladder afferents contain neuropeptides that can be released upon activation by physiological stimuli such as bladder wall distention or in response to mechanical injury, antigens, and bacterial or viral infection [15,30,31]. Peptides are also released from the urothelium into the LP [32]. A change in the local balance of peptides and other mediators (e.g., ATP), as it may occur as a result of neural or bladder mucosa injury or urinary bladder inflammation [16,31,33,34], can change bladder excitability to a hyper- or hypo-activity state.

To eliminate potential influences of the CNS or the systemic circulation on *local* mechanisms in the bladder LP and to gain direct access to the LP, we performed the present study in a decentralized ex vivo murine bladder model with intact urothelium and LP but no detrusor muscle [1,11,21,22]. As in our previous studies, we used eATP as a substrate because it has greater fluorescence properties than ATP, enabling small changes in substrate and product concentrations to be detected without assessing the endogenous adenyl purines. In agreement with our previous work [11,21,22], physiological filling of the detrusor-free bladder was accompanied by an increasing release of s-ENTDs in the LP. Consequently, the degradation of eATP in cELS of distended preparations exceeded the degradation of eATP in cELS of nondistended preparations. The degradation of eATP by spontaneously released s-ENTDs in the bladder LP was significantly increased in the presence of exogenous CGRP, Sub P, and NKA but not of exogenous PACAP (1-38), whereas the distention-induced release of s-ENTDs was increased by NKA and PACAP (1-38) but not by CGRP and Sub P. Therefore, neuropeptides differentially alter the constitutive and mechanosensitive release of s-ENTDs. All changes in s-ENTD release by exogenous neuropeptides were mediated by specific neuropeptide receptors. Thus, the effect of CGRP was inhibited by the competitive CGRP antagonist CGRP8-37 [23], which is an N-terminally truncated peptide that, like the agonist CGRP, has the highest binding affinity for the CGRP1 receptor. The latter is the result of heterodimerization of the calcitonin-like receptor (CLR) and the receptor activity-modifying protein 1 (RAMP1), CRL-RAMP1 [35,36]. The non-peptide CGRP receptor antagonist BIBN 4096 [37], however, failed to influence the degradation of eATP by s-ENTDs, likely because it acted on receptors that differ from those sensitive to CGRP8-37. Alternatively, it could be because BIBN 4096 has significantly greater affinity for human CGRP receptors than for rodent CGRP receptors [38]. The effects of Sub P on the degradation of eATP by spontaneously released s-ENTDs were inhibited by the non-competitive NK1 receptor inhibitor CP 122721 [24], whereas the NK2 receptor inhibitor MEN 10376 [25] diminished the effect of NKA on the distention-induced release of s-ENTDs. Finally, the PAC1 receptor antagonist PACAP (6-38) [26] inhibited the increasing effect of exogenous PACAP (1-38) on the degradation of ATP by spontaneously released s-ENTDs.

It has been shown that PAC1 receptors are selective for PACAP (1-38) and PACAP (1-27), whereas VPAC1 and VPAC2 receptors respond to both VIP and PACAP (1-38) with high affinity [39]. Notably, VIP binds primarily to VPAC receptors, as it has about one thousand times lower affinity for the PAC1 receptor than PACAP [40]. In the present study, exogenous PACAP (1-27), VIP, and the VPAC2 receptor agonist Bay 55-9837 [41] all failed to affect the spontaneous or distention-induced release of s-ENTDs and the consequent ATP degradation. These results, together with the data with PACAP (1-38) and PACAP (6-38), suggest that PAC1 but not VPAC1 or VPAC2 receptors are likely involved in the regulation of s-ENTD release in the mouse bladder LP. PAC1 receptors are expressed in bladder nerve fibers, urothelium, and the detrusor [42,43]. Involvement of PAC1 receptors in the modulation of s-ENTD release in the LP was also suggested by our earlier study using the same bladder model [21]. PACAP (1-27) is a C-terminal truncated isoform of PACAP (1-38) that can stimulate PAC1 and VPAC receptors. However, of the two PACAP isoforms, PACAP (1-38) is the predominant form in most tissues and organ systems [39]. This may be the reason why, in the present study, PACAP (1-38) but not PACAP (1-27) had an effect on the distention-induced release of s-ENTDs.

In aggregate, the results of the present study suggest that the activation of CGRP, NK1, NK2, and PAC1 receptors by exogenous neuropeptides affects in different ways the spontaneous and distention-induced release of s-ENTDs in the bladder LP. Thus, CGRP and NK1 receptors appeared to be involved in the regulation of the spontaneous release of s-ENTDs, the PAC1 receptor appeared to be involved in the regulation of the distention-induced release of s-ENTDs, and NK2 receptors seemed to be involved in the regulation of both the spontaneous and the distention-induced release of s-ENTDs upon exogenous application of peptides. Such mechanisms might be particularly important during bladder inflammation or spinal cord injuries when high amounts of neuropeptides or upregulation of neuropeptide receptors in the bladder wall occur [14,31,34,44,45]. Moreover, inflammation causes increased stretch-activated release of ATP by bladder urothelium, suggesting augmented purinergic signaling in the inflamed bladder [46,47]. Therefore, the facilitated degradation of extracellular ATP in response to increased activation of neuropeptide receptors might be an attempt at compensatory mechanisms to counteract the elevated extracellular ATP in response to pathophysiological stimuli. Employing redundant reactions and controls would ensure the proper excitability of target cells.

Of particular interest in the present study is the observation that, in a number of instances, the degradation of eATP was potentiated not only by exogenous neuropeptides but also by specific antagonists of neuropeptide receptors. Such results suggested that certain endogenous and exogenous neuropeptides likely have opposite effects on the release of e-ENTDs and the consequent degradation of ATP in the LP. For example, inhibition of CGRP receptors with CGRP8-37 and of PAC1 receptors with PACAP (6-38) both increased the distention-induced, but not the spontaneous, release of s-ENTDs, suggesting that endogenous CGRP and PACAP (1-38) might accomplish “controlled inhibition” of ATP hydrolysis at the end of bladder filling [21]. Inhibition of NK1 receptors with CP122721 increased both the spontaneous and distention-induced release of s-ENTDs in the LP, suggesting that Sub P (or other specific agonists of CP122721-sensitive receptors) is likely secreted in the LP spontaneously and during bladder filling and restrains the release of s-ENTDs and the extracellular hydrolysis of ATP. Unlike antagonists of CGRP, PAC1, and NK1 receptors, the NK2 inhibitor MEN 10376 had no effect on the degradation of extracellular ATP, although it inhibited the effects of exogenous NKA (Figure 6). These results suggest that NK2 receptors are likely present in the LP/urothelium of the mouse bladder, but endogenous activators of these receptors do not participate in the regulation of s-ENTD release in the LP. The discrepancy between the effects of exogenous and endogenous neuropeptides might be due to the activation of different receptor populations (e.g., junctional vs. extrajunctional receptors) by the corresponding agonists. Thus, the effect of endogenous neuropeptides is likely mediated by receptors that are located in close proximity to the site of release of neuropeptides (e.g., neuroeffector junction), whereas exogenously applied peptides likely stimulate a broader range of receptors that might also be located outside of the effector junction. It is also possible that the concentrations of endogenous and exogenous peptides at receptor sites differ, which might trigger different intracellular cascade mechanisms that depend on the strength of receptor activation. Although the mechanisms of regulated release of s-ENTDs in the bladder LP remain to be elucidated, it is possible that s-ENTDs are released by cell membrane/ectodomain shedding that might be regulated differently by diverse signaling pathways [48]. The type of ENTDs that are affected by individual neuropeptides cannot be determined at present due to the complexity of extracellular ATP metabolism by multiple s-ENTDs that are released simultaneously in the bladder LP during filling (e.g., ENTPD1 > ENTPD3 >> ENPP3 > ENPP1 = ENTPD2 = NT5E >> ENTPD8 = TNAP [11]) and frequently demonstrate overlapping substrate specificity [10].

The majority of neuropeptide receptors are coupled to Gα_s_-, Gα_i_-, Gα_q/11_, and/or Gα_12/13_ proteins [49,50,51], and therefore, multiple signaling pathways (e.g., phospholipase C-, adenylyl cyclase-, phospholipase A2-, and extracellular signal-regulated kinase 1/2 (ERK1/2)-activated) could be triggered. The presence, absence, or abundance of different neuropeptide receptors and their endogenous agonists may result in multiple outcomes and physiological and pathological consequences. Usually, the resultant predominant signaling is dependent on the relative expression levels and active conformation of the G proteins [52]. In some cases, when the neuropeptide binds to the corresponding receptor (e.g., PACAP binding to PAC1), the receptor is internalized through endocytosis, and endosomal signaling leads to diverse downstream effects [53,54]. The most studied intracellular signaling pathway in neuropeptide actions seems to be the activation of adenylyl cyclase and the generation of cAMP [35,54]. Clearly, however, activation of this pathway is not uniformly involved in the regulation of s-ENTD release. Further studies are warranted to unravel the signal transduction pathways that underlie the regulation of s-ENTD release in the bladder LP by neuropeptides.

In summary, the present study reinforces the notion that the effective concentrations of ATP and bioactive metabolites in the bladder LP are under intricate regulation. Release of s-ENTDs and ATP hydrolysis are increased during bladder filling, while mechanisms that restrain further release of s-ENTDs as the bladder filling progresses avert excessive degradation of ATP. The present study puts forward the novel idea that sensory neuropeptides play a role in the complex regulation of bladder excitability by controlling the extracellular degradation of ATP and the formation of bioactive purines deep in the bladder wall. Stimulation of neuropeptide receptors can generate various second messengers that can trigger a range of effector mechanisms, resulting in increased release of s-ENTDs in the bladder LP. Further studies are warranted to understand the interdependence of peptidergic and purinergic signaling mechanisms in the regulation of bladder function.

## 4. Materials and Methods

### 4.1. Animals

The Institutional Animal Care and Use Committee at the University of Nevada, Reno, approved all animal procedures in this study in accordance with the National Institutes of Health Guide for the Care and Use of Laboratory Animals. C57BL/6 mice (Jackson Laboratory, Bar Harbor, ME, USA; JAX stock# 000664) were housed and maintained in rooms with controlled temperature and humidity under 12-h light-dark cycles. Animals were provided with water and food ad libitum. When the animals reached 12–18 weeks of age, they were anesthetized with isoflurane and euthanized by cervical dislocation. The urinary bladders were quickly removed and placed in a cold Krebs bicarbonate solution (KBS) with the following composition (mM): 118.5 NaCl, 4.2 KCl, 1.2 MgCl_2_, 23.8 NaHCO_3_, 1.2 KH_2_PO_4_, 11.0 dextrose, and 1.8 CaCl_2_ (pH 7.4).

### 4.2. Ex Vivo Detrusor-Free Bladder Preparation

After harvesting the bladder, the detrusor muscle was removed as previously described [1,11,55]. Briefly, the bladder was placed in a Sylgard-covered dish filled with oxygenated cold KBS. The tissue was carefully pinned through the ureters, urethra, and apex of the serosa to the dissecting dish. The connective and fat tissues surrounding the bladder were removed using fine forceps and surgical scissors. Then, the detrusor was gently cut and separated from the suburothelium without pulling until the suburothelium/LP was completely exposed. The preparation was catheterized through the urethra using a PE-20 short tube, and the maximum capacity of the bladder was determined by filling the bladder through the catheter with a syringe filled with KBS.

### 4.3. Collection of Extraluminal Solutions Containing s-ENTDs

Detrusor-free bladder preparations were placed in 3 mL water-jacketed chambers containing KBS with either vehicle or drug, when appropriate. The solution in the chamber containing the bladder was oxygenated at all times with a gaseous mixture of 95% O_2_/5% CO_2_ and maintained at pH 7.4. The preparation was equilibrated for 20 min, after which the solution in the chamber was replaced with a fresh solution. Then, the bladder was left empty (nondistended condition) for a time equivalent to reaching voiding pressure, as determined at the time of bladder dissection. Next, 2.9 mL of the bath solution (called “extraluminal solution”, ELS) were collected and transferred to an Amicon centrifugal tube with a 10 kDa molecular weight cut-off (MWCO) pore size (Millipore Sigma, Burlington, MA, USA) for further processing as described in Section 2.4. Then, the chamber was filled with a fresh solution containing a drug or vehicle, and the bladder was filled through the catheter at a rate of 15 µL/min with KBS using a syringe pump (Kent Scientific, Torrington, CT, USA) to ~85-90% of its maximum capacity (distended condition). At the end of bladder filling, 2.9 mL of ELS were collected and processed the same way as the ELS obtained from nondistended preparations (see Section 2.4).

Bladder preparations were treated with solutions containing receptor antagonists during dissection, equilibration, and the actual experiment to ensure inhibition of receptor targets. Agonists were absent from the solutions used for tissue dissection and equilibration of bladder preparations to avoid unnecessary desensitization due to prolonged contact between tissue and receptor agonists.

### 4.4. Preparation of Reaction Solutions Containing Released s-ENTDs

The ELS samples that were obtained from nondistended and distended bladder preparations and placed in the ultra-centrifugal filter units were concentrated to a final volume of 80–100 µL at 4000× *g* for 25 min at 4 °C using a SorvallST 40R centrifuge (Thermofisher, Langenselbold, Germany). After centrifugation, the volume of the concentrated ELS (cELS) was transferred to a 0.6 mL Eppendorf tube and adjusted to a 200 µL reaction volume with oxygenated fresh KBS [11,21,22].

### 4.5. Time-Course of eATP Hydrolysis by s-ENTDs in cELS from Nondistended and Distended Bladder Preparations

To assess s-ENTD activities, the substrate 1,N^6^-etheno-ATP (eATP, final concentration 2 µM) was added to the reaction tube containing s-ENTDs. The reaction tube was kept at 37°C in a water bath, and following the start of the reaction, 20 µL samples were taken at 10’’, 2’, 4’, 6’, 8’, 10’, 20’, 30’, 40’, and 60’ and transferred to HPLC polyethylene inserts prefilled with 180 µL cold citric buffer to stop the enzymatic reactions, preserve the purines from spontaneous degradation, and dilute the samples 10-fold. The inserts were placed in HPLC glass vials and stored at −20 °C until further processing using HPLC methodology with fluorescence detection (HPLC-FLD) [11,55].

### 4.6. Chemical Synthesis of the 1,N^6^-Etheno-ATP (eATP) Substrate

eATP has approximately 1,000,000-fold higher fluorescence than authentic ATP [56]. The eATP substrate was prepared as described previously [1,11]. Citrate phosphate buffer was added to 0.2 mM ATP (Sigma-Aldrich, St. Louis, MO, USA) to acidify the medium to pH 4.0. Then, 2-Chloroacetaldehyde (1 M) was added to the solution, which was subsequently heated to 80°C for 40 min to synthesize 1,N^6^-etheno-ATP [56,57].

### 4.7. HPLC Analysis of 1,N^6^-Etheno-Nucleotides and 1,N^6^-Etheno-Nucleosides

The levels of the substrate eATP and its metabolites 1,N^6^-etheno-ADP (eADP), 1,N^6^-etheno-AMP (eAMP), and 1,N^6^-etheno-adenosine (eADO) in samples obtained from cELS from nondistended and distended preparations were measured using a reverse-phased gradient Agilent Technologies 1200 liquid chromatography system equipped with a fluorescence detector (Agilent Technologies, Wilmington, DE, USA) as described [1,11,56]. The areas of the peaks corresponding to etheno-purines were calculated and calibrated using standards containing known concentrations of eATP, eADP, eAMP, and eADO.

### 4.8. Drugs

From Tocris (Bio-Techne, Minneapolis, MN, USA): His-Ser-Asp-Ala-Val-Phe-Thr-Asp-Asn-Tyr-Thr-Arg-Leu-Arg-Lys-Gln-Val-Ala-Ala-Lys-Lys-Tyr-Leu-Gln-Ser-Ile-Lys-Asn-Lys-Arg-Tyr-NH2 (Bay 55-9837), 1-[3,5-Dibromo-*N*-[[4-(1,4-dihydro-2-oxo-3(2*H*)-quinazolinyl)-1-piperidinyl]carbonyl]-D-tyrosyl-L-lysyl]-4-(4-pyridinyl)-piperazine (BIBN 4096), CGRP, CGRP8-37, (2*S*,3*S*)-*N*-[[2-Methoxy-5-(trifluoromethoxy)phenyl]methyl]-2-phenyl-3-piperidinamine dihydrochloride (CP 122721), Asp-Tyr-{d-Trp}-Val-{d-Trp}-{d-Trp}-Lys-NH2 (MEN 10376), NKA, PACAP (1-38), PACAP (6-38), Sub P, and VIP. From Sigma-Aldrich (St. Louis, MO, USA): ATP, ADP, AMP, adenosine, and DMSO.

### 4.9. Statistical Analyses of the Results

Each chromatography peak was assessed by measuring the area under the peak and plotted against a standard curve. Data analyses were performed using Excel (Microsoft Corporation, Redmond, WA, USA) and GraphPad Prism v.8.4.2 (GraphPad Software, Inc., San Diego, CA, USA) software. Values are expressed as the mean ± SD. Data were considered statistically significant when comparisons yielded *p* values < 0.05. Asterisks were used to indicate statistical significance: * *p* < 0.05, ** *p* < 0.01, *** *p* < 0.001, **** *p* < 0.0001. A two-way analysis of variance (ANOVA) with Sidak’s *post hoc* analysis was used for multiple comparisons between time courses of enzymatic reactions. In addition, the area under the curve (AUC) for each experiment was calculated using GraphPad Prism as an integrated measurement, which computes the area using the trapezoid rule. AUC values ranged between 0 and 1, as both the fraction of total purines and time in hours ranged from 0 to 1. AUC values from two groups or more were compared by an unpaired t-test or an ordinary one-way ANOVA, respectively. A probability value less than 0.05 was considered statistically significant. Note that this is an exploratory study [58], hence the calculated *p*-values are descriptive.

## Data Availability

The raw data supporting the conclusion of this article will be made available by the authors upon reasonable request. The data are not publicly available due to privacy.

## References

[1] Durnin L., Kwok B., Kukadia P., McAvera R., Corrigan R.D., Ward S.M., Zhang Y., Chen Q., Koh S.D., Sanders K.M. (2019). An ex vivo bladder model with detrusor smooth muscle removed to analyse biologically active mediators released from the suburothelium. J. Physiol..

[2] Ferguson D.R., Kennedy I., Burton T.J. (1997). ATP is released from rabbit urinary bladder epithelial cells by hydrostatic pressure changes—A possible sensory mechanism?. J. Physiol..

[3] Matsumoto-Miyai K., Kagase A., Yamada E., Yoshizumi M., Murakami M., Ohba T., Kawatani M. (2011). Store-operated Ca^2+^ entry suppresses distention-induced ATP release from the urothelium. Am. J. Physiol. Renal Physiol..

[4] McLatchie L.M., Fry C.H. (2015). ATP release from freshly isolated guinea-pig bladder urothelial cells: A quantification and study of the mechanisms involved. BJU Int..

[5] Wang E.C., Lee J.M., Ruiz W.G., Balestreire E.M., von Bodungen M., Barrick S., Cockayne D.A., Birder L.A., Apodaca G. (2005). ATP and purinergic receptor-dependent membrane traffic in bladder umbrella cells. J. Clin. Investig..

[6] Merrill L., Gonzalez E.J., Girard B.M., Vizzard M.A. (2016). Receptors, channels, and signalling in the urothelial sensory system in the bladder. Nat. Rev. Urol..

[7] Dalghi M.G., Montalbetti N., Carattino M.D., Apodaca G. (2020). The Urothelium: Life in a Liquid Environment. Physiol. Rev..

[8] Burnstock G. (2014). Purinergic signalling in the urinary tract in health and disease. Purinergic. Signal..

[9] de Groat W.C., Griffiths D., Yoshimura N. (2015). Neural control of the lower urinary tract. Compr. Physiol..

[10] Zimmermann H., Zebisch M., Strater N. (2012). Cellular function and molecular structure of ecto-nucleotidases. Purinergic. Signal..

[11] Aresta Branco M.S.L., Gutierrez Cruz A., Dayton J., Perrino B.A., Mutafova-Yambolieva V.N. (2022). Mechanosensitive Hydrolysis of ATP and ADP in Lamina Propria of the Murine Bladder by Membrane-Bound and Soluble Nucleotidases. Front. Physiol..

[12] Gabella G. (2019). Afferent nerve fibres in the wall of the rat urinary bladder. Cell Tissue Res..

[13] Fowler C.J., Griffiths D., de Groat W.C. (2008). The neural control of micturition. Nat. Rev. Neurosci..

[14] Girard B.M., Wolf-Johnston A., Braas K.M., Birder L.A., May V., Vizzard M.A. (2008). PACAP-mediated ATP release from rat urothelium and regulation of PACAP/VIP and receptor mRNA in micturition pathways after cyclophosphamide (CYP)-induced cystitis. J. Mol. Neurosci..

[15] Arms L., Vizzard M.A. (2011). Neuropeptides in lower urinary tract function. Handb. Exp. Pharmacol..

[16] Shaffer A.D., Ball C.L., Robbins M.T., Ness T.J., Randich A. (2011). Effects of acute adult and early-in-life bladder inflammation on bladder neuropeptides in adult female rats. BMC Urol..

[17] Zheng G., Harms A.K., Tail M., Zhang H., Nimmo A., Skutella T., Kiening K., Unterberg A., Zweckberger K., Younsi A. (2023). Effects of a neurokinin-1 receptor antagonist in the acute phase after thoracic spinal cord injury in a rat model. Front. Mol. Neurosci..

[18] Holzer P. (1998). Neurogenic vasodilatation and plasma leakage in the skin. Gen. Pharmacol..

[19] Khera M., Somogyi G.T., Kiss S., Boone T.B., Smith C.P. (2004). Botulinum toxin A inhibits ATP release from bladder urothelium after chronic spinal cord injury. Neurochem. Int..

[20] Smith C.P., Vemulakonda V.M., Kiss S., Boone T.B., Somogyi G.T. (2005). Enhanced ATP release from rat bladder urothelium during chronic bladder inflammation: Effect of botulinum toxin A. Neurochem. Int..

[21] Aresta Branco M.S.L., Gutierrez Cruz A., Borhani Peikani M., Mutafova-Yambolieva V.N. (2023). Sensory Neurons, PIEZO Channels and PAC1 Receptors Regulate the Mechanosensitive Release of Soluble Ectonucleotidases in the Murine Urinary Bladder Lamina Propria. Int. J. Mol. Sci..

[22] Aresta Branco M.S.L., Gutierrez Cruz A., Peri L.E., Mutafova-Yambolieva V.N. (2023). The Pannexin 1 Channel and the P2X7 Receptor Are in Complex Interplay to Regulate the Release of Soluble Ectonucleotidases in the Murine Bladder Lamina Propria. Int. J. Mol. Sci..

[23] Chiba T., Yamaguchi A., Yamatani T., Nakamura A., Morishita T., Inui T., Fukase M., Noda T., Fujita T. (1989). Calcitonin gene-related peptide receptor antagonist human CGRP-(8–37). Am. J. Physiol..

[24] McLean S., Ganong A., Seymour P.A., Bryce D.K., Crawford R.T., Morrone J., Reynolds L.S., Schmidt A.W., Zorn S., Watson J. (1996). Characterization of CP-122,721; a nonpeptide antagonist of the neurokinin NK1 receptor. J. Pharmacol. Exp. Ther..

[25] Maggi C.A., Giuliani S., Ballati L., Lecci A., Manzini S., Patacchini R., Renzetti A.R., Rovero P., Quartara L., Giachetti A. (1991). In vivo evidence for tachykininergic transmission using a new NK-2 receptor-selective antagonist, MEN 10,376. J. Pharmacol. Exp. Ther..

[26] Robberecht P., Gourlet P., De Neef P., Woussen-Colle M.C., Vandermeers-Piret M.C., Vandermeers A., Christophe J. (1992). Structural requirements for the occupancy of pituitary adenylate-cyclase-activating-peptide (PACAP) receptors and adenylate cyclase activation in human neuroblastoma NB-OK-1 cell membranes. Discovery of PACAP(6-38) as a potent antagonist. Eur. J. Biochem..

[27] Smith-Anttila C.J.A., Morrison V., Keast J.R. (2021). Spatiotemporal mapping of sensory and motor innervation of the embryonic and postnatal mouse urinary bladder. Dev. Biol..

[28] Schueth A., Spronck B., van Zandvoort M., van Koeveringe G.A. (2017). Computer-assisted three-dimensional tracking of sensory innervation in the murine bladder mucosa with two-photon microscopy. J. Chem. Neuroanat..

[29] Andersson K.E., McCloskey K.D. (2014). Lamina propria: The functional center of the bladder?. Neurourol. Urodyn..

[30] Steinhoff M.S., von Mentzer B., Geppetti P., Pothoulakis C., Bunnett N.W. (2014). Tachykinins and their receptors: Contributions to physiological control and the mechanisms of disease. Physiol. Rev..

[31] Birder L.A., Kullmann F.A. (2018). Role of neurogenic inflammation in local communication in the visceral mucosa. Semin. Immunopathol..

[32] Birder L., Andersson K.E. (2013). Urothelial signaling. Physiol. Rev..

[33] Hammond T.G., Saban R., Bost K.L., Harris H.W., Kaysen J.H., Goda F.O., Wang X.C., Lewis F.C., Navar G.L., Campbell W.C. (2000). Substance P dependence of endosomal fusion during bladder inflammation. Am. J. Physiol. Renal Physiol..

[34] Ojala J., Tooke K., Hsiang H., Girard B.M., May V., Vizzard M.A. (2019). PACAP/PAC1 Expression and Function in Micturition Pathways. J. Mol. Neurosci..

[35] McLatchie L.M., Fraser N.J., Main M.J., Wise A., Brown J., Thompson N., Solari R., Lee M.G., Foord S.M. (1998). RAMPs regulate the transport and ligand specificity of the calcitonin-receptor-like receptor. Nature.

[36] Roehrkasse A.M., Booe J.M., Lee S.M., Warner M.L., Pioszak A.A. (2018). Structure-function analyses reveal a triple β-turn receptor-bound conformation of adrenomedullin 2/intermedin and enable peptide antagonist design. J. Biol. Chem..

[37] Doods H., Hallermayer G., Wu D., Entzeroth M., Rudolf K., Engel W., Eberlein W. (2000). Pharmacological profile of BIBN4096BS, the first selective small molecule CGRP antagonist. Br. J. Pharmacol..

[38] Rudolf K., Eberlein W., Engel W., Pieper H., Entzeroth M., Hallermayer G., Doods H. (2005). Development of human calcitonin gene-related peptide (CGRP) receptor antagonists. 1. Potent and selective small molecule CGRP antagonists. 1-[*N*^2^-[3,5-dibromo-*N*-[[4-(3,4-dihydro-2(1*H*)-oxoquinazolin-3-yl)-1-piperidinyl]carbonyl]-d-tyrosyl]-l-lysyl]-4-(4-pyridinyl)piperazine: The first CGRP antagonist for clinical trials in acute migraine. J. Med. Chem..

[39] Harmar A.J., Fahrenkrug J., Gozes I., Laburthe M., May V., Pisegna J.R., Vaudry D., Vaudry H., Waschek J.A., Said S.I. (2012). Pharmacology and functions of receptors for vasoactive intestinal peptide and pituitary adenylate cyclase-activating polypeptide: IUPHAR review 1. Br. J. Pharmacol..

[40] Vaudry D., Falluel-Morel A., Bourgault S., Basille M., Burel D., Wurtz O., Fournier A., Chow B.K., Hashimoto H., Galas L. (2009). Pituitary adenylate cyclase-activating polypeptide and its receptors: 20 years after the discovery. Pharmacol. Rev..

[41] Tsutsumi M., Claus T.H., Liang Y., Li Y., Yang L., Zhu J., Dela Cruz F., Peng X., Chen H., Yung S.L. (2002). A potent and highly selective VPAC2 agonist enhances glucose-induced insulin release and glucose disposal: A potential therapy for type 2 diabetes. Diabetes.

[42] Braas K.M., May V., Zvara P., Nausch B., Kliment J., Dunleavy J.D., Nelson M.T., Vizzard M.A. (2006). Role for pituitary adenylate cyclase activating polypeptide in cystitis-induced plasticity of micturition reflexes. Am. J. Physiol. Regul. Integr. Comp. Physiol..

[43] Girard B.M., Malley S.E., Braas K.M., May V., Vizzard M.A. (2010). PACAP/VIP and receptor characterization in micturition pathways in mice with overexpression of NGF in urothelium. J. Mol. Neurosci..

[44] Rapp D.E., Turk K.W., Bales G.T., Cook S.P. (2006). Botulinum toxin type a inhibits calcitonin gene-related peptide release from isolated rat bladder. J. Urol..

[45] Lucioni A., Bales G.T., Lotan T.L., McGehee D.S., Cook S.P., Rapp D.E. (2008). Botulinum toxin type A inhibits sensory neuropeptide release in rat bladder models of acute injury and chronic inflammation. BJU Int..

[46] Sun Y., Chai T.C. (2006). Augmented extracellular ATP signaling in bladder urothelial cells from patients with interstitial cystitis. Am. J. Physiol. Cell Physiol..

[47] Taidi Z., Mansfield K.J., Bates L., Sana-Ur-Rehman H., Liu L. (2019). Purinergic P2X7 receptors as therapeutic targets in interstitial cystitis/bladder pain syndrome; key role of ATP signaling in inflammation. Bladder.

[48] Lichtenthaler S.F., Lemberg M.K., Fluhrer R. (2018). Proteolytic ectodomain shedding of membrane proteins in mammals-hardware, concepts, and recent developments. EMBO J..

[49] Weston C., Winfield I., Harris M., Hodgson R., Shah A., Dowell S.J., Mobarec J.C., Woodlock D.A., Reynolds C.A., Poyner D.R. (2016). Receptor Activity-modifying Protein-directed G Protein Signaling Specificity for the Calcitonin Gene-related Peptide Family of Receptors. J. Biol. Chem..

[50] Sun J., Ramnath R.D., Tamizhselvi R., Bhatia M. (2008). Neurokinin A engages neurokinin-1 receptor to induce NF-kappaB-dependent gene expression in murine macrophages: Implications of ERK1/2 and PI 3-kinase/Akt pathways. Am. J. Physiol. Cell Physiol..

[51] Sánchez M.L., Rodríguez F.D., Coveñas R. (2023). Peptidergic Systems and Cancer: Focus on Tachykinin and Calcitonin/Calcitonin Gene-Related Peptide Families. Cancers.

[52] Reiter E., Ahn S., Shukla A.K., Lefkowitz R.J. (2012). Molecular mechanism of β-arrestin-biased agonism at seven-transmembrane receptors. Annu. Rev. Pharmacol. Toxicol..

[53] May V., Parsons R.L. (2017). G Protein-Coupled Receptor Endosomal Signaling and Regulation of Neuronal Excitability and Stress Responses: Signaling Options and Lessons from the PAC1 Receptor. J. Cell. Physiol..

[54] Alexander T.I., Tasma Z., Siow A., Rees T.A., Brimble M.A., Harris P.W.R., Hay D.L., Walker C.S. (2023). Novel Fluorescently Labeled PACAP and VIP Highlight Differences between Peptide Internalization and Receptor Pharmacology. ACS Pharmacol. Transl. Sci..

[55] Gutierrez Cruz A., Aresta Branco M.S.L., Perrino B.A., Sanders K.M., Mutafova-Yambolieva V.N. (2022). Urinary ATP Levels Are Controlled by Nucleotidases Released from the Urothelium in a Regulated Manner. Metabolites.

[56] Bobalova J., Bobal P., Mutafova-Yambolieva V.N. (2002). High-Performance Liquid Chromatographic Technique for Detection of a Fluorescent Analogue of ADP-Ribose in Isolated Blood Vessel Preparations. Anal. Biochem..

[57] Levitt B., Head R.J., Westfall D.P. (1984). High-pressure liquid chromatographic-fluorometric detection of adenosine and adenine nucleotides: Application to endogenous content and electrically induced release of adenyl purines in guinea pig vas deferens. Anal. Biochem..

[58] Vollert J., Schenker E., Macleod M., Bespalov A., Wuerbel H., Michel M., Dirnagl U., Potschka H., Waldron A.M., Wever K. (2020). Systematic review of guidelines for internal validity in the design, conduct and analysis of preclinical biomedical experiments involving laboratory animals. BMJ Open Sci..

